# Development of high-throughput genotyping method of all 18 HR HPV based on the MALDI-TOF MS platform and compared with the Roche Cobas 4800 HPV assay using clinical specimens

**DOI:** 10.1186/s12885-019-6036-z

**Published:** 2019-08-22

**Authors:** Xushan Cai, Qinghua Guan, Yu Huan, Ziyu Liu, Jiehua Qi, Shichao Ge

**Affiliations:** 1 Department of Clinical Laboratory, Jiading District Maternal and Children Health Hospital, No. 1216 Gaotai Road, Jiading District, Shanghai, 201899 People’s Republic of China; 2 Department of Research and Development, Shanghai Benegene Biotechnology Inc., Building 25, Pujiang Hi-tech Park, No. 588 Xinjunhuan Road, Minhang District, Shanghai, 201114 People’s Republic of China

**Keywords:** Human papillomavirus, MALDI-TOF MS platform, Recombinant plasmid, The Roche Cobas 4800 HPV assay, Genotyping

## Abstract

**Background:**

To develop a new 18 high-risk human papillomavirus (HR HPV) detection and genotyping assay, which is important to evaluate the risk degree of HR HPV for causing cancers.

**Methods:**

All 18 HR HPV and β-globin relative DNA fragments were synthesized and cloned to a plasmid pUC57 to obtain their recombinant plasmids. Based on the matrix-assisted laser desorption/ionization time-of-flight mass spectrometry (MALDI-TOF MS) platform, each of the 18 HR HPV genotypes were investigated using their constructed recombinant plasmids. The new 18 HR HPV genotyping assay was tested using 356 clinical specimens and the results were compared to ones detected by the Roche Cobas 4800 HPV assay (Cobas). The discrepant results between two assays were resolved by sequencing and genotyping methods.

**Results:**

The new 18 HR HPV MALDI-TOF MS genotyping assay was developed using HPV recombination plasmids. The sensitivity was 10^3^ to 10^2^ copies/reaction for the all 18 HR HPV. This new developed HR HPV genotyping test was used to detect the clinical specimens. When the results on clinical samples detected by the new MALDI-TOF MS HPV test were compared with ones detected by the Roche Cobas 4800 HPV assay in terms of 14 HR HPV, the concordance was 80.1% (kappa coefficient, 0.60; 95% confidence interval [CI], 0.52–0.69). The discrepant results were resolved by sequencing and genotyping and suggests that the developed HR HPV assay is more sensitive and specific.

**Conclusions:**

The new developed 18 HR HPV detection method based on MALDI-TOF MS platform is a high-throughput assay for the all 18 HR HPV genotypes and a powerful complement to current detection methods.

**Electronic supplementary material:**

The online version of this article (10.1186/s12885-019-6036-z) contains supplementary material, which is available to authorized users.

## Background

Cervical cancer is the second most common malignant cancer in women worldwide. Persistent infection with high-risk types of human papillomavirus (HR HPV) has been established as a necessary, but insufficient, factor in the development of high-grade cervical dysplasia and cervical carcinoma [1]. HPV DNA could be detected in 99.7% of cervical carcinoma [2]. In addition, HPV testing had a high negative predictive value for CIN2 (cervical intraepithelial neoplasia grade II) of greater than 99%, enabling screening intervals to be extended up to 5 years or longer [3]. Therefore, HPV DNA detection is of significance in the prevention and diagnosis of cervical cancer [4]. More than 100 HPV types have been identified and approximately 45 types can infect the genital tract [5]. Based on their oncogenic potential, HPV has been divided into 3 categories: 15 HR types including HPV16, 18, 31, 33, 35, 39, 45, 51, 52, 56, 58, 59, 68, 73 and 82; 3 probable HR including HPV26, 53 and 66; and low risk (LR) types such as HPV6, 11, 40, 42, 43, 44, 54, 61, 70, 72, 81, and CP6108 [1]. HR HPV are associated with neoplastic lesions and carcinomas, while LR HPV are mainly associated with benign lesions. The distribution of type-specific HPV varies by geographic region. HPV16 is the most prevalent HR HPV type everywhere in the world, HPV18 is slightly more prevalent in Europe and North America, HPV31 is more prevalent in South/Central America, HPV33 and 45 are more prevalent in Africa, and HPV52 and 58 are more prevalent in Asia, such as China and South Korea [6, 7].

The strong causal relationship between HR HPV and cervical cancer revealed that screening for HR HPV types is necessary for the prevention and control of cancers [2]. The Hybrid Capture II (HC2) HR HPV test (Qiagen, Inc., Valencia, CA) was approved in 2006 by the U.S. Food and Drug Administration (FDA) and widely used in clinical testing. However, it does not identify specific HPV types. Recently, other commercial HPV tests have become available. One of these is the Roche Cobas 4800 HPV test (Roche, Molecular Systems, Pleasanton, CA), which was approved by the FDA in 2011. The Roche Cobas 4800 HPV test was designed to amplify 14 HR HPV types including HPV16, 18, 31, 33, 35, 39, 45, 51, 52, 56, 58, 59, 66 and 68 [8]. This assay can identify HPV 16 and 18, but a pooled result for the remaining 12 HR HPV types. However, HPV genotyping plays a critical role in determining the prevalence and relative risk degree of each type of HPV, monitoring the recurrence after cancer treatment and evaluating the efficacy of prophylactic vaccines.

Multiplex polymerase chain reaction (PCR) together with the matrix-assisted laser desorption/ionization time-of-flight mass spectrometry (MALDI-TOF MS) platform (Sequenom, Inc., San Diego, CA) is a novel method for type specific detection of HR oncogenic HPV types. This assay contains a three-step process consisting of multiplex PCR, primer extension with a single nucleotide, and MALDI-TOF mass separation of extended products on a matrix-loaded silicon chip array. Based on the MALDI-TOF MS platform, a genotyping method of the 14 HR HPV types aforementioned in the Roche Cobas 4800 HPV assay kit was developed [9]. A 15 HR HPV genotyping method was also developed on the MALDI-TOF MS platform [10].

The aim of this study was to develop a detection method for genotyping of the 18 HR HPV types, namely all currently known HR and probable HR HPV types (16, 18, 26, 31, 33, 35, 39, 45, 51–53, 56, 59, 66, 68, 73, 82) based on the MALDI-TOF platform. The relative DNA fragment of each 18 HR HPV was firstly cloned into a plasmid pUC57 to obtain their recombinant plasmids, which was used as standard type templates, and a high-throughput HR HPV genotyping method was investigated. The effectiveness of this method was compared with a commercial kit, the Roche Cobas 4800 HPV assay, which detects 14 HR HPV types in a total of 356 cervical clinical specimens. Discrepant results between them were analyzed by sequencing and genotyping.

## Methods

### Construction of HPV recombinant plasmids

DNA L1 regions of all 18 HR HPV mentioned above and human β-globin DNA used as an internal control were searched out from the NCBI website (https://www.ncbi.nlm.nih.gov/) (see Additional file 1: Table S1). The relative DNA fragment of each 18 HR HPV was synthesized and cloned into the multiple cloning sites of pUC57 vector using restriction endonuclease *SmaI*. The recombinant pUC57-HPV DNA fragment plasmids were transformed into *Escherichia coli* DH5α and screened by the Blue-White plaque technique on Luria-Bertani (LB) agar plates containing 100 μg/ml of ampicillin [11]. These obtained bacterial strains containing recombinant HPV DNA plasmid each were cultured and plasmid DNA isolated. The copy number of recombinant plasmid containing viral DNA fragment per unit was calculated according to DNA concentration determined by using the NanoDrop Spetrophotometer (Thermo Scientific, Waltham, MA).

### Development of the 18 HR HPV genotyping assay using the established recombinant plasmids

The assay was designed for simultaneous detection and genotyping of 18 HR HPV types in two separated wells, one of which contained 9 HPV-plex and β-globin primer pairs. The multiplex PCR was performed in a total of 5 μl reaction volume with 2.5 mM MgCl_2_, 200 μM each dNTP, 200 pM of primer pairs mix, 0.1 U/μl HotStar Taq enzyme (Qiagen, Inc., Valencia, CA) and 1 μl of recombinant HPV plasmid DNA (1 ng/μl) using as templates in a 384 well plate format (Sequenom Inc., San Diego, CA). Water was included as a negative control in every test. The consensus primer pairs GP5+/GP6+ [12] with some modifications were used. The generic 10-mer tag ACGTTGGATG was added to the 5′ end of each primer, whose function is to make sure mass of the amplification primer being greater than the following extension primer and its extension products. The whole process including the multiplex PCR, a single-base extension and MALDI-TOF MS separation of products on a matrix-loaded silicon chip array was performed according to the manufacturer’s instructions (Sequenom Inc., San Diego, CA) [9, 13]. The extension primers were designed using three software programs: (1) Primer3 software (http://frodo.wi.mit.edu) [14] was applied to determine the location and sequence of the primers; (2) the OligoEvaluator™ (SIGMA-ALDRICH) internet software (http://www.oligoevaluator.com/OligoCalcServlet) was used to detect secondary structure and primer dimer itself; (3) the MassARRAY Assay Designer software 4.0 (Sequenom Inc., San Diego, CA) was used to check whether the formation of primer dimer among different extension primers or not in the same well. Sequences and molecular weights of extended primers were listed in Additional file 2: Table S2.

### Clinical specimens collection

A total of 356 clinical samples used for this study were from women visiting the gynecology outpatient clinics of Jiading District Maternal and Children Health Hospital of Shanghai, China. The median age of the patients was 39.8 years (range from 22 to 68 years old). According to the protocols of practice, the cervicovaginal cells at the transformation zone of the uterine cervix were collected by a gynecologist or a trained gynecologist assistant with a standard cytobrush (with spatula), and suspended in PreservCyt® (Hologic Inc., Bedford MA, USA).

### Clinical specimens HPV genotyping by the 18 HR HPV MALDI-TOF MS assay

The collected samples in PreservCyt medium for routine liquid-based cytology (LBC) were processed in a room of the laboratory physically separated from where the PCR amplification was performed. Briefly, 2 ml of cellular liquid in PreservCyt medium was removed into an Eppendorf tube for sedimentation. Then the gravity-sedimentary cellular material was lysed in a 400 μl of digestion solution containing 5ul of proteinase at 65 °C for 30 min, followed by treatment in 150 μl NaCl solution. The DNA in the lysed supernatant was precipitated by water-free alcohol, and the pellets were dissolved in TE buffer. The clinical DNA specimens were detected and genotyped using the developed 18 HR HPV MALDI-TOF MS assay. To avoid cross-contamination, the laboratory spaces were separated into three parts: the rooms for pre-PCR processes (DNA extraction, quantification and gel electrophoresis), for PCR processes (PCR reaction system preparation and reactions), and for post-PCR processes (PCR product extensions and conducts of mass spectrometry), respectively.

### HPV detection by the Roche Cobas 4800 HPV assay

The Roche Cobas 4800 HPV test was carried out according to the manufacturer’s protocol for detection of 14 HR HPV genotypes including HPV16, 18, 31, 33, 35, 39, 45, 51, 52, 56, 58, 59, 66, and 68 (Roche, Molecular Systems, Pleasanton, CA) [8]. Briefly, DNA was extracted via a fully automated sample preparation process using the cobas × 480 instrument and samples was transferred to a cobas z 480 analyzer for PCR amplification of the 14 HR HPV DNA. The sequences of approximately 200 nucleotides were produced within the highly conserved L1 region of the HPV genome and fluorescent oligonucleotide probes specific for real-time detection of individual HPV16, HPV18, and the 12 other HR HPV genotypes were pooled. The human β-globin gene (330-bp amplicon) was included as an internal control to provide a measure of specimen adequacy as well as to monitor the quality of extraction and amplification process, and positive and negative controls were included in each run. Interpretation of the amplification and detection was carried out using software supplied with the Roche Cobas 4800 HPV assay.

### HPV genotyping by direct sequencing

For the samples with discrepant results between two HPV detection tests, their HPV genotypes were identified using PCR and sequencing. Nested PCR method was performed by using the general primer pair MY09/11 for primary PCR and the GP6+/MY11 or GP6+/GP5+ primer pairs for nested PCR [15]. The visualized PCR amplicons were sequenced by direct automated fluorescent dye-terminator Sanger method using GP6+ nucleotide as the sequencing primer. The genotype-specific sequence was validated through online BLAST algorithms. When more than one HPV types was present in a sample, type specific primer pairs were used for identification of multiple HPV infection as our previous study [15]. All positive bands of type-specific nested PCR amplicons during gel electrophoresis were purified and sequenced using one of the genotype-specific primers as the sequencing primer. The obtained sequences were aligned to the GenBank database using the BLAST server and HPV types were identified when the identity was equal or more than 95%. The human β-globin gene was also simultaneously tested as an internal control for specimen integrity.

### Statistical analysis

Concordance was calculated assuming a standard 2 × 2 contingency table [16]. The degree of agreement between the MALDI-TOF MS and the Cobas 4800 test in detecting HR HPV genotypes was assessed using the Cohen’s kappa statistics, with values of 0.00 to 0.20 indicating slight agreement, 0.21 to 0.40 fair agreement, 0.41 to 0.60 moderate agreement, 0.61 to 0.80 substantial agreement, and 0.81 to 1.0 almost perfect agreement [13]. The 95% confidence interval (95% CI) was calculated in the Cohen’s kappa values assuming a binomial distribution. Marginal homogeneity of the two tests was assessed by a two-sided McNemar test. The value of *p* < 0.05 was considered significant. Discordant samples were analyzed in sequencing and genotyping using the pearson χ^2^-test.

## Results

### Development of the 18 HR HPV MALDI-TOF MS genotyping assay using their recombinant plasmids

**Fig. 1:**
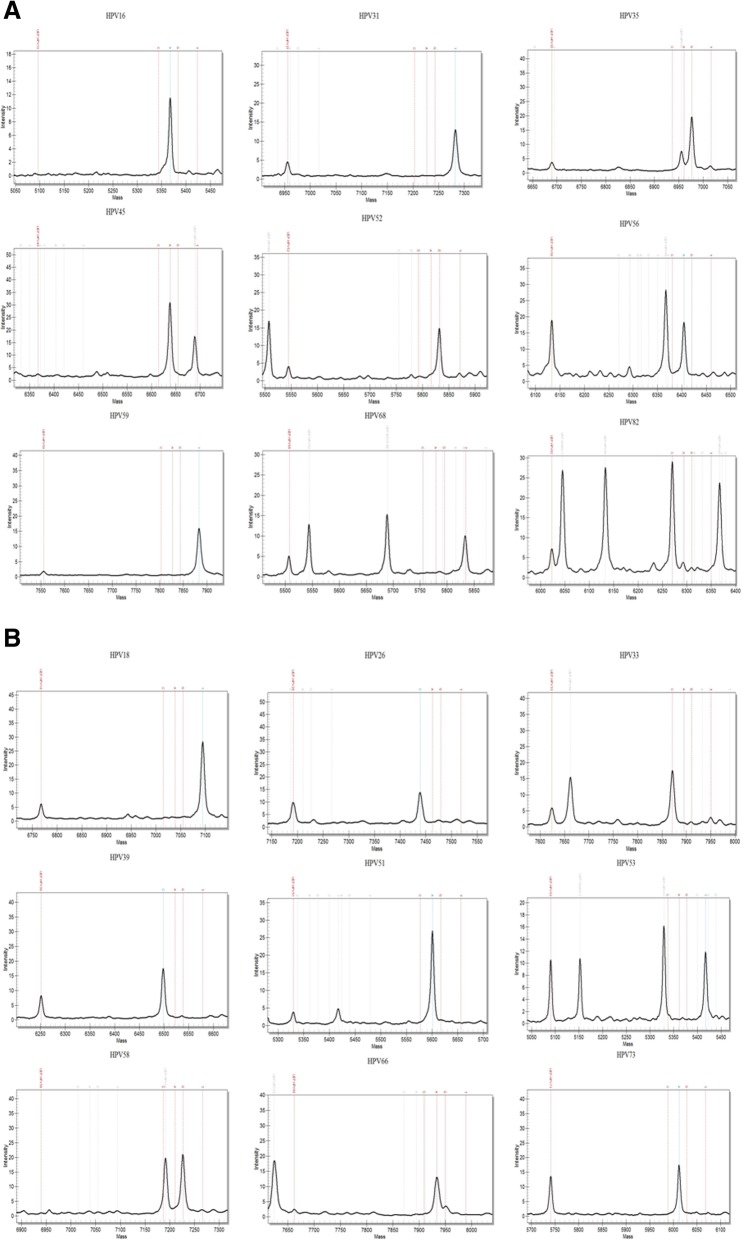
The 18 HR HPV genotyping assay using their recombination plasmids as templates during multiplex PCR based on the MALDI-TOF MS platform. **a** The 9 panels showed the results of 9 HR HPV types in one well. **b** The 9 panels showed the results of the other 9 HR HPV types in another well. The x-axis of each panel depicted the molecular weights of the extended products and its unextended primer which were showed as peaks, and the peak representing an unextended primer present suggested it was not used up in the extension reactions. The y-axis depicts the intensity of each peak. Certain panels contained other unextended primer peaks since its weight is in the range of the panel detecting the HPV genotype

To develop the 18 HR HPV genotyping assay, each of their relative HPV DNA was synthesized and cloned into a plasmid pUC57 to obtain their recombination plasmids. The obtained 18 HR HPV DNA recombinant plasmids were sequenced to confirm the existence of each of HPV DNA fragments. Next, multiplex PCR reactions were carried out using their HPV recombinant plasmids as templates based on the MALDI-TOF MS platform. Each type-specific assay was optimized and then all the 18 HR HPV primers were combined into a two-well reaction. Each of the 9-plex HPV including HPV16, 31, 35, 45, 52, 56, 59, 68 and 82 was identified in one well (Fig. 1a), and each of the other 9-plex HPV including HPV18, 26, 33, 39, 51, 53, 58, 66 and 73 in another one (Fig. 1b). The new developed genotyping assay could identify each of all the 18 HR HPV in a two-well reaction based on the MALDI-TOF MS platform.

To determine the sensitivity, serial ten-fold dilutions of the recombinant plasmids containing HPV DNA were tested. These dilutions were ranged from 10^6^ to 10 copies. The detection limit was 10^3^ for HPV 26, 33, 45 and 56, and10^2^ copies for HPV 16, 18, 31, 35, 39, 51–53, 58, 59, 66, 68 73 and 82. The results suggested that the sensitivity ranged from 10^3^ up to 10^2^ copies/reaction for all 18 HR HPV types.

For the specificity**,** the MALDI-TOF MS HPV assay using the 18 HR HPV recombinant plasmids as template could identify one or two target peaks, representing an extension product and an unextended primer in certain types, and all were found without cross-reaction (Fig. 1).

### Detection of clinical samples by the 18 HR HPV MALDI-TOF MS assay

The new developed 18 HR HPV MALDI-TOF MS assay was used to test the clinical specimens for their prevalence and genotyping. This assay allowed detection and genotyping of clinical samples in two wells of one reaction. A 24-h laboratory could provide a throughput of 4500 samples per day with current configuration [9]. Among a total of 356 clinical specimens tested, 171 samples were positive including 141 single infection and 30 multiple infections (Table 1). The positive rate was at 48.0% (171/356) for all clinical specimens. The five most common HR HPV types were HPV16 being 24.4% (87/356), HPV58 9.83% (35/356), HPV52 7.58% (27/356), HPV33 3.65% (13/356), and HPV53 2.80% (10/356). A single HPV genotype accounted for 39.6% (141/356), whereas multiple types were 8.43% (30/356) in all the samples. Among the latter, 5.90% (21/356) had dual infections, 2.53% (9/356) had triple infections or more.

**Table 1:** Prevalence and genotype of HPV for clinical specimens by the 18 HPV MALDI-TOF MS assay

Single infection	No of samples	Multiple infections	No of samples
HPV16	66	HPV16/33	2
HPV18	7	HPV16/52	3
HPV31	2	HPV16/53	1
HPV33	10	HPV16/58	4
HPV35	2	HPV16/66	3
HPV39	1	HPV16/68	1
HPV45	1	HPV33/45	1
HPV51	2	HPV52/58	5
HPV52	13	HPV56/66	1
HPV53	8	HPV16/52/56	1
HPV56	3	HPV16/52/58	2
HPV58	19	HPV16/53/68	1
HPV59	1	HPV16/58/68	2
HPV66	3	HPV52/58/66	1
HPV68	2	HPV16/52/58/59	1
HPV73	1	HPV18/52/58/68	1
Subtotal	141		30

### Comparison between the 18 HR HPV MALDI-TOF MS assay and the Roche Cobas 4800 HPV assay on clinical samples

All of the 356 samples were tested by the Roche Cobas 4800 assay, and the positive rate of these samples was 44.4% (158/356) and slightly less than the positive rate of 48.0% which was produced by MALDI-TOF MS assay. In terms of 14 HR types, which could be detected by the Roche Cobas 4800 HPV assay, the concordance between them was 80.1% (kappa coefficient, 0.60; 95% CI, 0.52–0.69) (Table 2). For HPV16 genotype, the results for both assays were in substantial agreement with each other (kappa coefficient, 0.68, concordance rate = 88.5%). For HPV 18, the concordance rate between the results was 98.9% with kappa 0.71. For the other 12 HR HPV types, which can be detected together by the Roche Cobas 4800, the agreement between two assays was 82.6% (kappa coefficient, 0. 53).

**Table 2:** Concordance between the results of the 18 HPV MALDI-TOF MS and the Roche Cobas 4800 assays

HPV genotype	PCR-MS results	Results of Cobas assay			Concordance rate (%)	Kappa coefficient	95% CI
		Positive	Negative	Total			
All	Positive	129	42	171	80.1	0.60	0.52–0.69
	Negative	29	156	185			
	Total	158	198	356			
16	Positive	65	22	87	88.5	0.68	0.59–0.77
	Negative	19	250	269			
	Total	84	272	356			
18	Positive	5	3	8	98.9	0.71	0.17–0.94
	Negative	1	347	348			
	Total	6	350	356			
Other 12 HR HPV^a^	Positive	57	32	89	82.6	0.53	0.40–0.62
	Negative	30	237	267			
	Total	87	269	356			

^a^including HPV 31, 33, 35, 39, 45, 51, 52, 56, 58, 59, 66 and 68

### Detection of discrepant results between the 18 HR HPV MALDI-TOF MS assay and the Roche Cobas 4800 assay by sequencing and genotyping

For the discrepant results between the MALDI-TOF MS HPV test and the Roche Cobas 4800 HPV assay, sequencing and genotyping was used to identify HPV types. For the 22 HPV16 which were positive only by MALDI-TOF MS assay, 16 (72.7%) of them were identified as HPV16 by sequencing and genotyping, the remaining 6 samples were identified as 2 HR HPV including 52 and 82, and 5 LR HPV including HPV6, 11, 54 and 81, and 2 negative (Table 3). The types were more than samples because some samples contained more than one HPV type. While 12 of 19 (63.2%) specimens which were HPV 16 positive only by the Roche Cobas 4800 HPV were positive upon sequencing and genotyping, the remaining 7 samples were identified as 3 HR HPV and 6 LR HPV. For the 3 samples which were HPV18 positive by MALDI-TOF MS but negative by the Roche Cobas 4800 assay, sequencing and genotyping confirmed 2 of them (66.7%) as HPV18, while for the one positive HPV18 samples only by the Roche Cobas 4800, none was confirmed as HPV18. Among the 32 samples which were positive only by the MALDI-TOF MS assay in terms of other 12 HR types, 20 were confirmed in detection by sequencing and genotyping, and 6 of 7 HPV53 and 1 HPV73 high-risk types identified, which are not detectable in Cobas 4800 test, were also confirmed. The MALDI-TOF MS assay was cross-reaction with LR HPV types including HPV6(2), 40(2), 54(1), 81(2) samples. For the 30 samples which were positive only by the Roche Cobas 4800 test, 16 were positive by sequencing and genotyping. In the remaining 14 samples, most of them were identified as LR HPV, including HPV6(2), 11(2), 40(1), 42(2), 43(1), 44(1), 71(1), 72(2), and 81(3) (Table 3). These results suggested that the new developed 18 HR HPV MALDI-TOF MS assay was less cross-reactive with LR HPV than the Roche Cobas 4800 HPV assay.

**Table 3:** Resolving on discrepance between the 18 HPV MALDI-TOF MS and the Roche Cobas 4800 assays by sequencing

HPV genotype	Disagreement of both assays	Results of sequencing and genotyping on dis-concordant results of both assays			Conc. rate (%)	Results of sequencing and genotyping on dis-concordance
		Conc.	Dis-conc.	Total		
HPV16	MALDI-TOF MS+/Cobas-	16	6	22	72.7	6 (1), 11 (1), 52 (1), 54 (1), 81 (2), 82 (1), neg(2)
	MALDI-TOF MS−/Cobas+	12	7	19	63.2	6 (2), 40 (1), 42 (1), 43 (2), 52 (2), 58 (1), neg(2)
HPV18	MALDI-TOF MS+/Cobas-	2	1	3	66.7	neg(1)
	MALDI-TOF MS−/Cobas+	0	1	1	0	58 (1)
Other 12 HR HPV	MALDI-TOF MS+/Cobas-	20	12	32	62.5	6 (2), 40 (2), 53 (7), 54 (1), 73 (1), 81 (2), neg(3)
	MALDI-TOF MS−/Cobas+	16	14	30	53.3	6 (2), 11 (2), 16 (4), 40 (1), 42 (2), 43 (1) 44 (1), 53 (2), 71 (1), 72 (1), 81 (3), 82 (2), neg(4)

*Conc.* Concordance, *dis-conc.* Disconcordance, *neg* Negative

## Discussion

Persistent infection of HR HPV can lead to high-grade pre-cancer and cervical cancer [17]. It is therefore important to identify type-specific HPV for clinical application and prevent cancer [18]. Based on MALDI-TOF MS platform, using the constructed pUC57 recombinant plasmids as standard types in multiplex PCR, a new genotyping method for all the known 18 HR HPV was developed. To our knowledge, it was the first time for detection of the all known 18 HR HPV genotypes on the MALDI-TOF MS platform.

Using recombination plasmids containing HPV DNA, one group developed 14 HR HPV genotyping method including HPV16, 18, 31, 33, 35, 39, 45, 51, 52, 56, 58, 59, 66, and 68 based on the MALDI-TOF mass spectrometry platform [9]. The 15 HR HPV (HPV16, 18, 31, 33, 35, 39, 45, 51, 52, 56, 58, 59, 66, 68 and 73) genotyping method developed by using competitors for these HPV types based on the platform was more sensitive than PreTect HPV-Proofer assay for type-specific detection of the five most common oncogenic HPV including HPV16, 18, 31, 33 and 45 [10]. In another study, 16 HR HPV recombinant plasmids which contain inserts along E6 and E7 genes of HPV16, 18, 31, 33, 35, 39, 45, 51, 52, 56, 58 and 59, and the same kind of insert of HPV53, 66, 68 and 73 were constructed and assessed for development of the MALDI-TOF MS-based HPV assay [13]. The present study developed the detection and genotyping method for all currently known 18 HR HPV including 15 HR HPV and 3 probable HR HPV. This detection method was a fully automated high-throughput one, with a process capacity of 10 × 384-well format within 2 working days [19] compared with the Roche Cobas 4800 HPV assay having an ability to detect 14 HR HPV genetypes with individual genotyping of HPV 16 and 18 and a pooled result for the remaining 12 HR genotypes [8]. Besides, during this study, we found that it was very easy to produce cross-reactions among different HPV types, especially with increased numbers of multiplex PCR primers in one well. Therefore, we divided the 18 HR HPV types into two wells in one reaction, which greatly reduced the chance of cross-reactions among them.

The concordance was substantial agreement between the new developed MALDI-TOF MS 18 HR HPV genotyping method and the Roche Cobas 4800 HPV assay for clinical samples HR HPV detection in terms of 14 HR HPV types detected by the later. The Roche Cobas 4800 HPV test detects only HPV16, HPV18 and 12 other HR HPV as a pooled result, which does not identify individual HPV genotype. The new developed MALDI-TOF MS HPV assay can identify each of 18 HR HPV individually, meaning that it has more range of HR HPV detection spectrum than the Roche Cobas 4800 assay. Moreover, the sensitivity in HPV detection by MALDI-TOF MS was superior to that of real-time fluorescence PCR-based assays including the Roche Cobas 4800 assay in the present study and the previous report [20].

Sequencing has the ability to detect all known HPV types. Discrepant results between the new MALDI-TOF MS and the Roche Cobas 4800 HPV assays in detecting clinical samples were analyzed by sequencing and genotyping. In the MALDI-TOF MS−/Cobas+ samples, LR HPV including HPV 6, 11, 40, 42–44, 71, 72, and 81 were found, while in the MALDI-TOF MS+/Cobas- samples HPV 6, 11, 54 and 81 were seen by sequencing and genotyping. Much more LR HPV genotypes were found in Cobas-positive samples compared with MALDI-TOF MS-positive ones. The consequence of LR HPV genotype cross-reactivity in the Roche Cobas 4800 HPV test caused an increased number of false-positive results, leading to over-treatment for women who possessed only LR HPV genotype. This suggested that the new developed MALDI-TOF MS HPV test demonstrated much less cross-reactivity than the Roche Cobas 4800 test. Taken together, this MALDI-TOF MS HR HPV assay is an evolving tool with exciting potential to study the epidemiology and clinical management of HPV-associated diseases [21]. The cross-reactivity of HPV 42, 54, 61, 70 in the Roche Cobas 4800 test was observed in a previous study identified by Linear Array (LA) HPV genotyping test [22] though the performance of the Roche Cobas was equivalent to LA HPV test for HR HPV detection reported in another study [23].

HPV genotype distribution has been shown to vary by race and geographic region [7, 24]. A meta-analysis of HPV prevalence in 5 continents displayed that HPV 16 and HPV 18 were the most frequent types worldwide, and they account for approximately 70.9% of cervical cancers [25, 26]. However, the situation was different in China. This study showed that HPV16, 58 and 52 were the first three most prevalent types, which was consistent with our previous report [15] and other investigations in Chinese women [27–29]. It is HPV52 and 58 that are more prevalent following HPV 16 in China. Besides, HPV52 and HPV58 are also more prevalent in the other Asian countries compared to other regions of world [30, 31]. The high prevalence of HPV52 and 58 may be a challenge to cervical screening and prevention of HR HPV for Chinese women.

## Conclusions

This study demonstrated that a two-well assay was developed to detect and type all 18 HR HPV based on the MALDI-TOF MS platform. The new developed method was able to provide more exact individual HPV type information compared to the Roche Cobas 4800 HPV assay. Other advantages of the MALDI-TOF MS method were the high-throughput detection of clinical samples in 384-well plate format and readily amenable to automation, with more sensitivity and specificity.

## Additional files

Additional file 1: **Table S1.** The sequences of the 18 HR HPV L1 regions. (XLSX 20 kb)

Additional file 2: **Table S2.** The sequences of the 18 HR HPV extended primers. (DOCX 36 kb)

## Data Availability

The raw data generated in this study are available from the corresponding author on reasonable request.

## Abbreviations

CI: Confidence interval
CIN: Cervical intraepithelial neoplasia grade
HC2: Hybrid Capture II
HPV: Human papillomavirus
HR: High risk
LBC: Liguid-based cytology
LR: Low risk
MALDI-TOF MS: Matrix-assisted laser desorption/ionization time-of-flight mass spectrometry
PCR: Polymerase chain reaction
