# Supplementary material for: Development of high-throughput genotyping method of all 18 HR HPV based on the MALDI-TOF MS platform and compared with the Roche Cobas 4800 HPV assay using clinical specimens
Source: BMC Cancer. 2019 Aug 22;19:825. doi: 10.1186/s12885-019-6036-z (PMC6704492; doi:10.1186/s12885-019-6036-z)
Supplement: Supplementary file 2 — Table S2. The sequences of the 18 HR HPV extended primers. (DOCX 36 kb) [file 12885_2019_6036_MOESM2_ESM.docx]

**Table S2** The **s**equences and molecular weights of the 18 HR HPV extended primers as well as a single base extended

| HPV | Extension primers (5→3) | Length of amplicons | Mass of products | Single base extended |
| --- | --- | --- | --- | --- |
| 16  18  26  31  33  35  39  45  51  52  53  56  58  59  66  68  73  82 | AAATCATATTCCTCCCC  GGGCAATATGATGCTACCAAAT  CATCTGCATCCACTCCATTTAAAC  CAATTGCAAACAGTGATACTACA  CAGTACTAATATGACTTTATGCACA  TGTGTTCTGCTGTGTCTTCTA  CCAACTTTACCTTATCTACCT  ACAAAATCCTGTGCCAAATAC  TTCCCCAACATTTACTCC  CATGACTTTATGTGCTGA  CCTCTGCATGCCTAACA  TGATGCACGAAAAATTAATC  ATGTACCTTCCTTAGTTACTTCA  CTGTGTGTGCTTCTACTACTTCTTC  GATTGATTTCACGGGCATCATATTT  ACATGCCTAATATATTCC  ACAACGTATGCCAACTCTA  CAGACATTCACTCCAACAAA | 17  23  25  24  27  22  22  22  19  19  18  21  24  26  26  19  20  21 | 5367.6  7094.6  7339.0  7282.8  7871.3  6687.4  6498.4  6638.5  5600.8  5831.9  5417.5  6404.3  7226.8  7883.1  7933.3  5834.8  6012.1  6270.3 | A  T  C  T  C  G  C  A  A  A  T  A  G  T  A  A  A  C |
